# Changes in physical activity patterns of students from primary to secondary school: a 5-year longitudinal study

**DOI:** 10.1038/s41598-022-15523-w

**Published:** 2022-07-04

**Authors:** Kanzo Okazaki, Yuzo Koyama, Kazunori Ohkawara

**Affiliations:** 1 Department of Human Science, Faculty of Liberal Arts, Tohoku Gakuin University, 2-1-1 Tenjinzawa Izumi-ku, Sendai, Miyagi 981-3193 Japan; 2 Seikei Junior and Senior High School, 3-10-13 Kichijoji-Kitamachi, Musashino-shi, Tokyo, 180-8633 Japan; 3 Graduate School of Informatics and Engineering, University of Electro-Communication, 1-5-1 Chofugaoka, Chofu, Tokyo 182-8585 Japan

**Keywords:** Lifestyle modification, Preventive medicine, Paediatric research, Epidemiology, Weight management

## Abstract

This study aimed to annually examine anthropometric characteristics and physical activity (PA) among children transitioning from fourth grade (9–10 years of age) to eighth grade (13–14 years of age) in Japan. The participants’ (*n* = 63) age, grade, sex, weight, height, and weight status were recorded at baseline. Accelerometry assessments were performed annually following established protocols. Time spent on activities requiring ≥ 3 metabolic equivalents (METs), 1.6–2.9 METs, and ≤ 1.5 METs were defined as moderate-to-vigorous intensity PA (MVPA), light PA (LPA), and sedentary behavior (SB), respectively. Two linear mixed models were used to examine whether MVPA, LPA, and SB min/day changed over time. Fifty-five participants provided complete data. A significant, nonlinear, longitudinal decline in MVPA, an increase in SB, and a linear decline in LPA were observed. Changes in MVPA and SB were accelerated in seventh grade (12–13 years of age). Male sex and maintenance of normal weight status were associated with higher MVPA levels. Changes in LPA and SB were not associated with sex or weight status. During the transition period from primary to secondary school, MVPA and SB showed nonlinear accelerations. Sex and normal weight were associated with more time spent performing MVPA.

## Introduction

A physically active lifestyle is beneficial for the physical and mental health of children and adolescents because it prevents or ameliorates obesity and coronary heart disease, enhances health-related quality of life, alleviates depression, and improves academic achievement^1,2^. With recent technological advancements, physical activity (PA) in terms of PA intensity in daily life can be estimated with devices such as accelerometer, which has become a common assessment tool^3^. Device-measured PA is typically classified as moderate-to-vigorous intensity PA (MVPA), light-intensity PA (LPA), and sedentary behavior (SB). MVPA comprises activities that generally require significant movement and energy expenditure (≥ 3.0 metabolic equivalents [METs]), such as brisk walking or running. LPA refers to casual walking, household chores, or activities of daily living (1.6–2.9 METs). SB refers to activities such as sitting that involve minimal movement and energy expenditure (≤ 1.5 METs)^4^. Since LPA is a part of daily activities, the interest in ensuring a healthy lifestyle among children and adolescents has shifted to MVPA and SB. Guidelines regarding MVPA^5^ and SB^6,7^ for children and adolescents are similar worldwide. In a pooled analysis of 298 population-based surveys involving 1.6 million students, those within 11–17 years of age showed a global trend toward insufficient PA based on current guidelines^8^.

In this study, we focused on the changes in PA during the transition from primary to secondary school, which is a critical developmental period and a turning point that may positively or negatively influence a child’s social and physical environment^9,10^. In Japan, students study in primary school until sixth grade and move to secondary school from the seventh grade. Long-term investigations with frequent data collection are necessary to gain a deeper understanding of the changes in PA that may occur during this transition. Recent systematic reviews have established that the period between childhood and adolescence is characterized by a decline in MVPA and an increase in SB^11,12^. However, in a systematic review by Chong et al.^11^, only six articles met their inclusion criteria for longitudinal and observational studies that reported repeated PA measurements, of which four measured MVPA, and only one study measured SB objectively with short (≤ 2 years) follow-up periods and few data collection points (in the first and/or second year of secondary school). The other systematic review^12^ analyzed only changes in SB and reported that overall daily sedentary time increased during the transition from primary to secondary school. However, only four studies used objective measurement methods, and one study used three or more time points for data collection. Furthermore, another recent systematic review^13^ reported changes in SB during childhood and adolescence over time. However, since this study covered students aged 5–18 years, these observations might not reflect changes in students from elementary to middle school. Consequently, objectively collected data of the PA transition from primary to secondary school are required to clarify long-term changes in PA patterns.

Although LPA constitutes part of the daily PA of children and adolescents, it has received much less research attention. Recent cross-sectional studies have shown that the proportion of device-measured LPA comprised 26%^14^ and 31%^15^ of all PA. The proportions of LPA in these prior studies were higher than those of MVPA. Evaluation of changes in LPA, as well as those in MVPA and SB, may be useful for the effective promotion of health among youths because LPA activities are part of daily activities and have the potential to enhance health^16,17^. However, little is known about LPA changes that occur during a child’s transition from primary to secondary school.

In light of the aforementioned topics, we aimed to examine potential changes in device-measured PA (MVPA, LPA, and SB) among children who transitioned from primary school (starting from fourth grade, 9–10 years of age) to secondary school (until eighth grade, 13–14 years of age) using annually collected longitudinal data. Our investigation also evaluated whether PA changes were linear or nonlinear. Our underlying rationale was that if a change involved a quadratic curve, health-promoting interventions focusing on the specific grade, wherein an accelerated change in PA was identified, may be considered.

## Results

### Participants

Table 1 shows the descriptive statistics of all participants based on their responses at each wave of the survey. The mean age (± standard deviation [SD]) of the initial 63 participants was 9.9 ± 0.3 years, and 56% were female. Fifty-five participants (87%) responded and provided PA data during each annual wave over the five-year study period. Eight participants were lost to follow-up because they had changed schools. Approximately 85% of the participants had normal weight status at the first wave, and this was maintained during the study.

**Table 1:** Descriptive statistics of participants (n = 55) in each assessment of this five-year longitudinal study on physical activity in children transitioning from primary to secondary school.

	Assessment 1	Assessment 2	Assessment 3	Assessment 4	Assessment 5
School grade	4th	5th	6th	7th	8th
Age, years (mean ± SD)	9.9 ± 0.3	10.9 ± 0.3	12.0 ± 0.2	12.9 ± 0.3	13.9 ± 0.3
**Sex**, **n (%)**					
Female	31 (56)	31 (56)	31 (56)	31 (56)	31 (56)
Male	24 (44)	24 (44)	24 (44)	24 (44)	24 (44)
Height, cm (mean ± SD)	140.0 ± 6.6	146.2 ± 7.0	152.6 ± 7.4	158.7 ± 7.0	162.6 ± 6.8
Weight, kg (mean ± SD)	33.2 ± 7.5	37.3 ± 8.9	41.6 ± 8.6	46.5 ± 8.5	50.8 ± 8.5
**Weight status**^**a**^**, n (%)**					
Underweight	6 (11)	6 (11)	6 (11)	2 (4)	4 (7)
Normal	46 (84)	45 (82)	46 (84)	48 (87)	48 (87)
Obese	3 (5)	4 (7)	3 (5)	5 (9)	3 (5)

*SD* standard deviation.

^a^Weight status refers to the percentage of overweight, calculated as the ratio of weight to the expected standard weight according to sex, age, and height.

### Changes in PA in the linear mixed model (LMM)

Table 2 shows the mean ± SD of the accelerometer data. At each assessment, the following number of participants did not satisfy the criterion of valid device-wearing for at least 600 min/day: assessment 1 (*n* = 3), assessment 2 (*n* = 1), assessment 3 (*n* = 3), assessment 4 (*n* = 2), and assessment 5 (*n* = 3).

**Table 2:** Accelerometer data from participants who met the device-wearing criterion^a^ in this five-year longitudinal study of physical activity in children transitioning from primary to secondary school.

Variable	Assessment 1 (n = 52)	Assessment 2 (n = 54)	Assessment 3 (n = 52)	Assessment 4 (n = 53)	Assessment 5 (n = 52)
**Daily physical activity**, **min (mean ± SD)**					
MVPA	67 ± 20	67 ± 29	71 ± 24	65 ± 28	53 ± 23
LPA	325 ± 51	296 ± 57	321 ± 54	286 ± 53	271 ± 57
SB	418 ± 82	453 ± 83	455 ± 101	502 ± 98	512 ± 114
Wearing time, min, (mean ± SD)	810 ± 81	815 ± 98	846 ± 120	853 ± 115	836 ± 132

Assessments 1, 2, 3, 4, and 5 took place in grades 4, 5, 6, 7, and 8, respectively.

*MVPA* moderate-to-vigorous intensity physical activity, *LPA* light-intensity physical activity, *SB* sedentary behavior, *SD* standard deviation.

^a^The device-wearing criterion required that the accelerometer be worn for at least 600 min/day during each assessment.

Tables 3 and 4 show the results of the LMM analyses using restricted maximum likelihood (REML) methods without (LMM I) and with (LMM II) adjustments for the fixed effect of sex and the interaction between sex and grade, respectively. A significant decrease in MVPA and an increase in SB were observed with a curvilinear pattern. The regression coefficients (β) of grade^2^ in LMM I (Table 3) were − 2.3 (95% confidence interval [CI], − 3.7 to − 0.9; *P* = 0.002) for MVPA and 3.3 (95% CI 0.4–6.2; *P* = 0.027) for SB, and those in LMM II (Table 4) were − 2.3 (95% CI − 3.7 to − 0.9; *P* = 0.002) for MVPA and 3.3 (95% CI 0.4–6.3; *P* = 0.025) for SB. A statistically significant linear decrease in LPA was observed in LMM I (β of grade = − 10.1; 95% CI − 19.8 to − 0.3; *P* = 0.043), whereas LPA in LMM II demonstrated a statistically insignifcant linear decrease (*P* = 0.063).

**Table 3:** Linear mixed models using the restricted maximum likelihood method without adjustments for the fixed effect of sex and the interaction between sex and grade in this five-year longitudinal study on physical activity in children transitioning from primary to secondary school.

Variable	MVPA			LPA			SB		
	Estimate (SE)	*t*	*P*-value	Estimate (SE)	*t*	*P*-value	Estimate (SE)	*t*	*P*-value
**Fixed part**									
Intercept	10.08 (13.41)	0.75	0.453	101.07 (24.64)	4.10	**0.000**	− 114.60 (31.67)	− 3.62	**0.000**
Grade	5.73 (2.98)	1.93	0.055	− 10.08 (4.95)	− 2.04	**0.043**	4.12 (6.29)	0.65	0.513
Grade^2^	− 2.25 (0.71)	− 3.17	**0.002**	− 1.03 (1.16)	− 0.89	0.374	3.31 (1.48)	2.23	**0.027**
**Weight status**									
Underweight	12.99 (8.73)	1.49	0.138	− 11.24 (16.08)	− 0.70	0.485	− 4.85 (19.86)	− 0.24	0.807
Normal	20.43 (7.08)	2.88	**0.004**	− 7.56 (13.16)	− 0.57	0.566	− 13.74 (16.12)	− 0.85	0.395
Obese	[Ref.]			[Ref.]			[Ref.]		
Accelerometer wear time	0.05 (0.01)	3.23	**0.001**	0.28 (0.02)	11.36	**0.000**	0.68 (0.03)	22.64	**0.000**
**Random effect**									
**Group**									
Intercept	17.72 (41.07)			84.70 (198.60)			298.51 (529.08)		
**Subjects (unstructured)**									
Grade	5.18 (9.80)			52.14 (31.55)			64.44 (48.41)		
Intercept	185.33 (83.10)			1330.71 (396.49)			2038.15 (621.55)		
Grade, intercept	6.88 (22.80)			− 73.25 (91.17)			− 169.66 (147.25)		
Residual	354.45 (41.31)			936.07 (109.19)			1531.70 (177.37)		

Estimated values of the fixed and random effects are reported by the regression coefficient and variance, respectively. Group effect describes the two groups recruited in grade 4 in 2015 (n = 32) and 2016 (n = 31), with differences at the time of baseline data collection.

Significant values are in bold.

*MVPA* moderate-to-vigorous physical activity, *LPA* light physical activity, *SB* sedentary behavior, *SE* standard error, *Ref* reference category.

**Table 4:** Linear mixed models using the restricted maximum likelihood method with adjustments for the fixed effect of sex and the interaction between sex and grade in this five-year longitudinal study on physical activity in children transitioning from primary to secondary school.

Variable	MVPA			LPA			SB		
	Estimate (SE)	*t*	*P*-value	Estimate (SE)	*t*	*P*-value	Estimate (SE)	*t*	*P*-value
**Fixed part**									
Intercept	21.31 (13.56)	1.57	0.117	95.72 (26.17)	3.66	**0.000**	− 121.18 (33.57)	− 3.61	**0.000**
Grade	4.08 (3.10)	1.32	0.189	− 9.80 (5.25)	− 1.87	0.063	5.40 (6.64)	0.81	0.417
Grade^2^	− 2.26 (0.71)	− 3.20	**0.002**	− 1.04 (1.16)	− 0.90	0.370	3.34 (1.48)	2.25	**0.025**
Female sex	− 18.85 (4.90)	− 3.85	**0.000**	10.99 (11.97)	0.92	0.359	7.51 (15.05)	0.50	0.618
Interaction between sex and grade	3.05 (1.79)	1.70	0.090	− 0.45 (3.45)	− 0.13	0.897	− 2.52 (4.21)	− 0.60	0.550
**Weight status**									
Underweight	14.39 (8.39)	1.72	0.087	− 11.75 (16.16)	− 0.73	0.468	− 4.38 (20.00)	− 0.22	0.827
Normal	21.18 (6.80)	3.12	**0.002**	− 7.57 (13.20)	− 0.57	0.566	− 13.42 (16.21)	− 0.83	0.409
Obese	[Ref.]			[Ref.]			[Ref.]		
Accelerometer wear time	0.04 (0.01)	3.10	**0.002**	0.28 (0.02)	11.10	**0.000**	0.69 (0.03)	22.20	**0.000**
**Random effect**									
**Group**									
Intercept	12.67 (31.37)			89.28 (205.18)			296.86 (529.26)		
**Subjects (unstructured)**									
Grade	4.16 (4.35)			54.80 (32.31)			66.91 (49.33)		
Intercept	100.10 (52.13)			1335.02 (401.93)			2080.88 (635.93)		
Grade, intercept	20.41 (8.07)			− 76.87 (92.87)			− 173.60 (150.26)		
Residual	353.51 (35.31)			936.44 (109.29)			1532.09 (177.48)		

Estimated values of the fixed and random effects are reported by the regression coefficient and variance, respectively. Group effect described the two groups recruited in grade 4 in 2015 (n = 32) and 2016 (n = 31), with differences at the time of baseline data collection.

Significant values are in bold.

*MVPA* moderate-to-vigorous physical activity, *LPA* light physical activity, *SB* sedentary behavior, *SE* standard error, *Ref* reference category.

**Figure 1:**
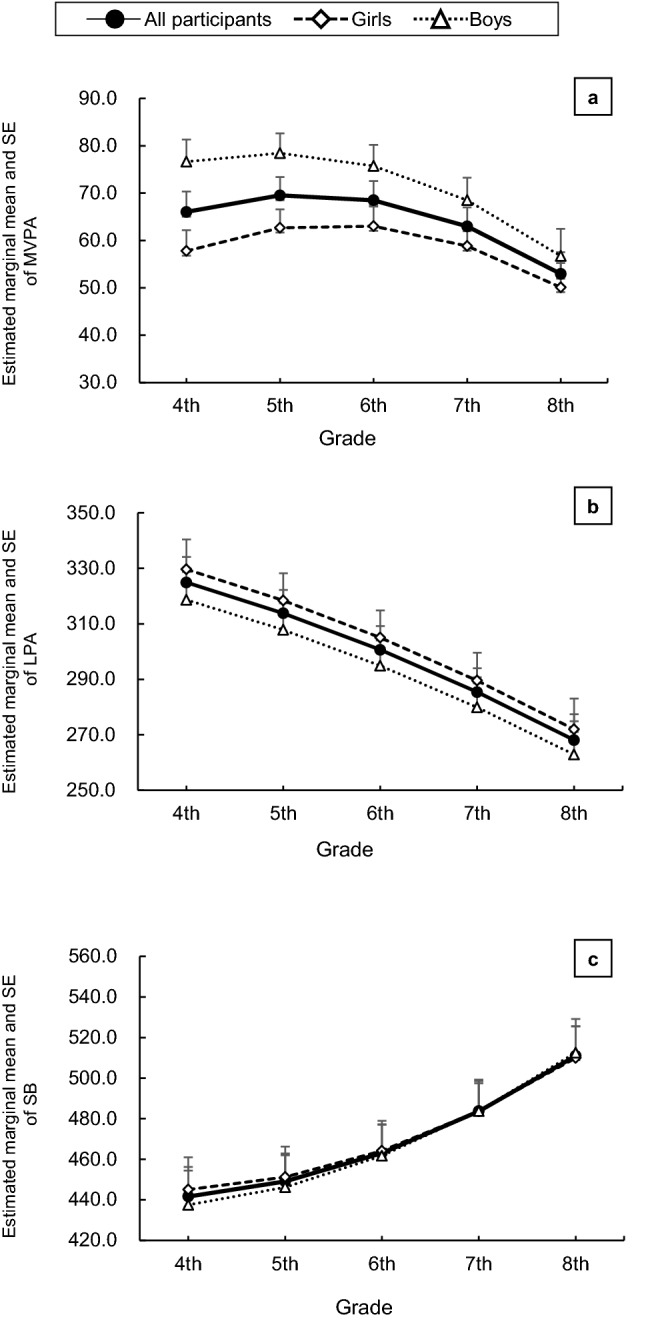
Quadratic trends for the different types of physical activity in linear mixed models I and II. (**a**) Moderate-to-vigorous intensity physical activity (MVPA); (**b**) light physical activity (LPA); (**c**) sedentary behavior (SB). ChAnges are expressed as estimated marginal means. The trends in linear mixed model I (solid line) were adjusted for body fat and accelerometer wearing time. The trends in linear mixed model II (dashed line and dotted line indicating girls and boys, respectively) were determined by adding the variables sex and the interaction between sex and grade. *SE* standard error.

Figure 1 shows the longitudinal quadratic trends of the estimated marginal means of MVPA, LPA, and SB in LMMs I and II. MVPA declined at an accelerated rate after seventh grade in girls and after sixth grade in boys (Fig. 1a). SB showed an accelerated increase after sixth grade in both sexes (Fig. 1c). The linear change in LPA was different from the curvilinear changes observed in MVPA and SB (Fig. 1b).

**Figure 2:**
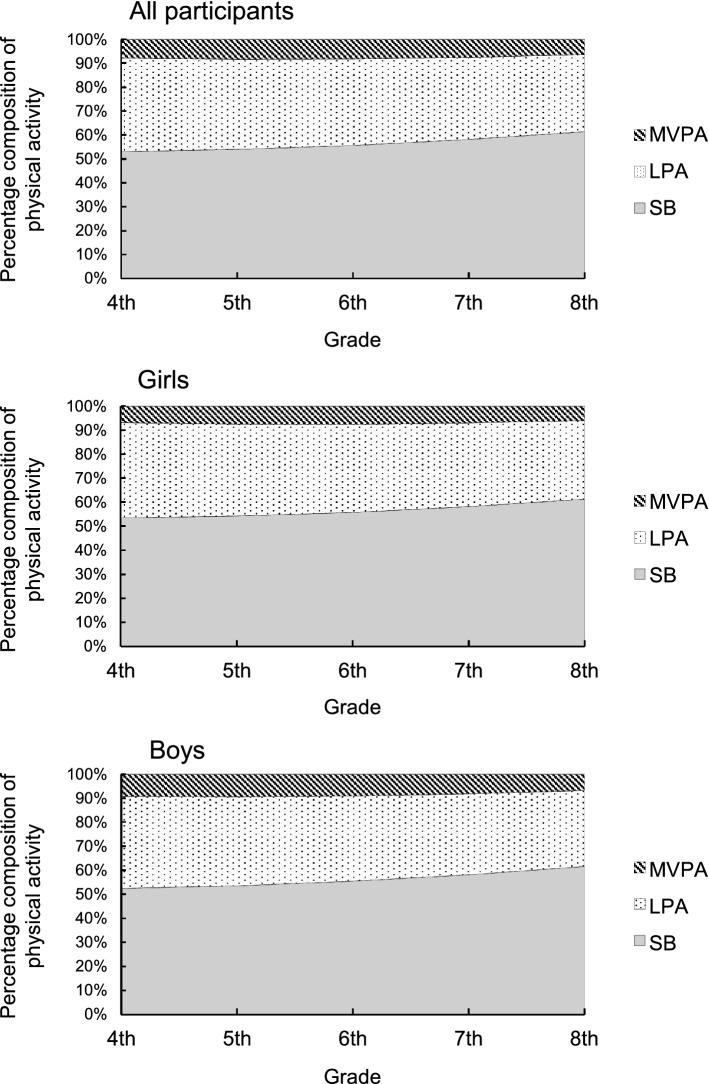
Composition of physical activity at each accelerometric assessment. The percentage composition is based on the estimated marginal means of MVPA, LPA, and SB in linear mixed models I (all participants) and II (girls and boys). *MVPA* moderate-to-vigorous intensity physical activity, *LPA* light-intensity physical activity, *SB* sedentary behavior.

A sex-related main effect in MVPA was observed (β = − 18.9; 95% CI − 28.5 to − 9.2; *P* < 0.001). The time spent performing MVPA was higher among boys than among girls, and there was no interaction between sex and grade (Table 4). When compared to obese children, normal-weight children had a significant influence on the increase of MVPA in LMM I (Table 3; β = 20.4; 95% CI 6.5–34.4; *P* = 0.004) and in LMM II (Table 4; β = 21.2; 95% CI 7.8–34.6; *P* = 0.002). However, the percentage of overweight (POW) had no influence on the changes in LPA and SB. Figure 2 shows the composition of habitual PA based on the estimated marginal means of MVPA, LPA, and SB at each assessment. The range of SB changes (53–62%) was similar to that of LPA changes (32–40%), whereas the range of MVPA changes was smaller (6–8%) over time.

## Discussion

The results of this longitudinal study showed that the decline in MVPA and increase in SB followed nonlinear functions in children transitioning from primary to secondary school (fourth to eighth grade, 9–14 years), whereas the decline in LPA was linear. This corresponded to an accelerated change in MVPA and SB among secondary school adolescents. Male sex and the maintenance of normal weight status over the 5 years were associated with higher levels of MVPA. However, the changes in LPA and SB over time were statistically unrelated to sex and weight status.

Our key finding of a decline in MVPA was consistent with the recent results reported by Farooq et al.^18^, who examined longitudinal PA data at ages 7, 9, 12, and 15 years in the Gateshead Millennium Cohort Study in England. A distinct linear trajectory based on the estimated marginal MVPA means derived from their LMM was detected upon adjustment for sex, age, and the interaction between these factors. Unlike these findings, our study demonstrated a nonlinear MVPA trajectory. Although the MVPA of girls in the study by Farooq et al. was less than the currently recommended 60 min/day^5^ after 9 years of age and continued to decline afterward, the girls in our study showed a slight increase in MVPA until 12 years of age. Moreover, there was an accelerated decline in MVPA when both boys and girls advanced from primary to secondary school.

Several longitudinal studies have examined PA changes using an accelerometer in children transitioning from primary to secondary school^11,12^. A current systematic review^11^, which included studies from 1990 to 2019, found no further studies reporting SB data since the 2017 review by Pearson et al.^12^. In a longitudinal study similar to ours, Mitchell et al.^19^ examined changes in SB (using a quantile regression model) at ages 9, 11, 12, and 15 years in children transitioning from primary to secondary school in the United States. A nonlinear increase in daily SB was detected in each year, with no sex-specific SB changes over time. Our results provided further support for a nonlinear, sex-independent increase in SB in children transitioning from primary to secondary school. Moreover, the SB increase observed in our study was underpinned by more continuous data and details. We observed a substantial increase when children transitioned to secondary school (after sixth grade). Compared to the findings of Mitchell et al.^19^, our results revealed a slower SB increase over time; this may be explained by the baseline 442 min/day of SB in our cohort, which was higher than the 320 min/day in their study. At ages 14 and 15 years, the gap in SB between our cohort (511 min/day) and the cohort reported by Mitchell et al. (492 min/day) was considerably narrower. However, our results were comparable to other current studies as they did not reflect a higher level of SB time among school-aged children^20,21^.

While the time spent performing LPA reportedly decreased as children grew into adolescents, few studies examined whether this LPA change was linear or nonlinear^19,22–24^. The LPA decrease in our cohort showed that adolescents in eighth grade spent 55 min less per day performing LPA than they did in fourth grade. This difference was smaller than that reported in a recent study based on pooled data from Europe, the United States, Brazil, and Australia from the International Children’s Accelerometry Database^22^. The pooled data (*n* = 1088) showed an 85-min decrease in LPA (from 360 to 275 min) among children aged 12–15 years. In addition, another longitudinal study^21^ indicated an 86-min decrease in LPA (from 287 to 201 min) among 75 Spanish children aged 8.5–13.8 years.

In our cohort, this LPA decrease was linear over the five years with annual data collection, whereas the MVPA and SB changes were nonlinear. Thus, we conclude that the linear decline in LPA in children began in primary school. The increased time spent in SB corresponded to the decrease in LPA in our analysis, indicating that time spent performing LPA was replaced by time spent in SB as children grew. Future studies should determine whether interventions focused on LPA may prevent an increase in SB.

Our results showed a temporal relationship between obesity and the changes in MVPA and SB. This finding is consistent with the conclusions from a previous systematic review by Elmesmari et al.^25^, who concluded that accelerometer-measured MVPA for obese children and adolescents was less than that for non-obese children aged 10–19 years without marked differences in SB. In contrast, a previous longitudinal study in children aged 9–15 years indicated a possible association between time spent in SB and the body mass index (BMI)^19^. Subjects in the 90th BMI percentile showed that the time spent in SB was associated with a curvilinear increase in BMI. However, there were weak or non-existent associations between SB duration and BMI for individuals in the 10th, 25th, and 50th BMI percentiles. Although there was no significant difference in SB duration between non-obese and obese participants in our cohort, negative estimates were shown in the LMM, implying that less time was spent in SB among non-obese participants than among obese participants. Whether obesity is affected by an increase in time spent in SB as children advance from primary to secondary school needs further investigation since our cohort included few obese children. An overview of interventions for preventing or treating pediatric and adolescent obesity found conflicting results on whether a decrease in BMI could be achieved by reducing SB time^26^.

Our results need to be interpreted within the limitations of this study. First, this study was conducted in a limited geographic area in Tokyo, Japan. Further studies are required to establish whether our findings can be replicated in more diverse populations. Second, although our findings were adjusted for covariates, residual confounding factors might have remained. Environmental, socioeconomic, and sociocultural confounding factors^27–29^ were not considered in the analysis. For example, since the study subjects attended private schools, tuition fees were higher than those of public schools. This schooling environment could be associated with parental income and availability of athletic facilities. Third, accelerometer could not record activity, while participants performed aquatic activities and did not detect the full scale of movement when the participant was cycling^30^. Fourth, children did not maintain a record of the periods during which they wore the accelerometer. Hence, their activities might have been underestimated. Additionally, we did not examine for reactivity^31^. However, the accelerometry methods, epoch length, non-wear time, and valid wearing minutes and days used in this study are widely used and accepted in child and adolescent populations^32^.

In summary, we found that the time spent in MVPA, LPA, and SB changed as children transitioned from primary to secondary school. The strengths of this study include the identification of linear and nonlinear changes with repeated objective annual data collection at five time points in a cohort of children progressing from fourth to eighth grade. MVPA and SB showed accelerated changes after the transition to secondary school, whereas changes in LPA were linear. In addition, we found that MVPA changes may be associated with obesity among children and adolescents; however, our cohort included few obese children.

## Methods

### Study design and participants

This longitudinal study was conducted at a private primary school and secondary school located in adjacent facilities in Tokyo, Japan. Data were annually collected (in either February or March) from 2015 to 2020. The two study groups were as follows: 1) the group that was initially surveyed in 2015 and longitudinally followed up every year between February and March for 5 years (n = 32) and 2) the group that was initially surveyed in 2016 and longitudinally followed up every year between February and March for 5 years (n = 31). This study began with baseline data collection of children in fourth grade (9–10 years of age) in 2015 (group 1, *n* = 32) and 2016 (group 2, *n* = 31). The children attended the same class, which was randomly selected by the school administration. We administered annual surveys to these children over five consecutive years until they reached eighth grade (secondary school; 13–14 years of age). A total of 63 healthy children (56% girls) provided baseline data in 2015 and 2016 and were tracked over the five-year study period.

Before the start of the study and at each follow-up survey and data collection point, parents received complete information about the purpose and methods of the study through their children’s teachers. Parental written consent was returned to the teachers after the participants’ parents had an opportunity to consider their child’s participation. All procedures involving human participants were performed in accordance with the ethical standards of the institutional and/or national research committee and with the 1964 Helsinki declaration and its later amendments or comparable ethical standards. The study protocol was approved by the Ethics Committee of the Graduate School of Human Informatics Study, Tohoku Gakuin University (Reference number 2017R001). Only children with parental written informed consent for participation were included.

### Measurements

#### Demographics and anthropometrics

Each participating child’s age, grade, sex, body weight, and body height were obtained using a self-administered questionnaire. Weight status, defined as the percentage of overweight (POW), was assessed using Japanese cutoff values based on the national reference data for Japanese children^33^. Briefly, POW is commonly utilized to assess childhood obesity in medical settings and schools in Japan and is calculated as the ratio of weight to the standard weight based on sex, age, and height. The POW criteria of obesity and being underweight are ≥ 20% and ≤ − 20%, respectively, with the normal range being -20% to 20%. These obesity and underweight criteria have been shown to correspond to age-adjusted BMI percentiles of > 86–89% and < 2–6%, respectively^33^.

#### PA measurements

The habitual PA of participants was assessed using a triaxial accelerometer (HJA-750C Active style Pro, Omron Healthcare, Kyoto, Japan) measuring 40 × 52 × 12 mm and weighing 23 g, including batteries. This triaxial accelerometer gathers information on the time spent on ambulatory and non-ambulatory activities of varying intensities. The accuracy of this device has been reported in prior studies^34,35^. Based on default predictive equations established for adults and the results of a prior study in children^36^, the following conversion equations were used. Ambulatory activities were calculated as 0.6237 × MET value + 0.2411, and non-ambulatory activities were calculated as 0.6145 × MET value + 0.5573. The time spent on activities requiring ≥ 3 METs, 1.6–2.9 METs, and ≤ 1.5 METs were considered as MVPA, LPA, and SB, respectively. We used the macro program (ver. 190829) developed and distributed by the Japan Physical Activity Research Platform (http://paplatform.umin.jp) to process the accelerometer data.

The accelerometry methods, including epoch length, non-wear time, and valid wearing minutes and days, were defined based on previous studies^32,37^. Participants were asked to wear the accelerometer on their waist for ≥ 7 consecutive days during all waking hours, except while showering, bathing, or swimming. The accelerometers were set to record data during 10-s sampling intervals (epochs) throughout the entire wearing period. The non-wearing time within a day was defined as any period with > 10 min of consecutive zero counts. Valid accelerometry data included > 600 min/day for minimum of four days, including one non-school day, were analyzed at each data collection point.

### Statistical analyses

We used LMM with REML methods to examine whether daily MVPA, LPA, and SB times changed during the five-year transition from primary school to secondary school. Based on prior evidence, the model included the school grade (time-coded: 0, 1, 2, 3, and 4) and grade^2^ (time-coded: 0, 1, 4, 9, and 16) in each wave of yearly time, sex, POW, and daily time spent wearing the accelerometer device as independent covariates in the fixed part of the model^19^.

We considered two models of LMM, LMM I and II, which were run without and with adjustments for the fixed effects of sex and the interaction between sex and grade, respectively. These models allowed a random effect within the group where the initial assessment time differed. We also fitted the models with the random effect of time-coded school grade in each participant. A quadratic trend was used to estimate the periods during which the MVPA, LPA, and SB had changed. All valid data at each collection time point were included in the analyses because LMMs are robust to missing data and can estimate longitudinal trends with incomplete datasets^38^. LMMs were used because they are robust to missing data and can estimate longitudinal trends with incomplete datasets. This method allowed us to include all valid data collected at each time point in the analyses. A total of 52, 54, 52, 53, and 53 participants were analyzed in assessments 1, 2, 3, 4, and 5, respectively, of this five-year longitudinal study (Table 2).

All statistical analyses were performed using Stata for Windows version 15.1 (Stata, College Station, TX, USA). All significance tests were two-sided, and the results were considered statistically significant at *P* < 0.05.

### Ethical approval

All procedures performed in studies involving human participants were in accordance with the ethical standards of the institutional and/or national research committee and with the 1964 Helsinki declaration and its later amendments or comparable ethical standards. The study design was approved by the Ethics Committee of the Graduate School of Human Informatics Study, Tohoku Gakuin University (Reference number 2017R001).

### Informed consent

Informed consent was obtained from the parents of all individual participants included in the study.

## Data Availability

The datasets generated during and/or analyzed during the current study are available from the corresponding author on reasonable request.

## References

[1] Wu XY (2017). The influence of physical activity, sedentary behavior on health-related quality of life among the general population of children and adolescents: A systematic review. PLoS ONE.

[2] Carson V (2016). Systematic review of sedentary behaviour and health indicators in school-aged children and youth: An update. Appl. Physiol. Nutr. Metab..

[3] Skender S (2016). Accelerometry and physical activity questionnaires: A systematic review. BMC Public Health.

[4] Ainsworth BE (2011). 2011 compendium of physical activities: A second update of codes and MET values. Med. Sci. Sports Exerc..

[5] Chaput JP (2020). 2020 WHO guidelines on physical activity and sedentary behaviour for children and adolescents aged 5–17 years: Summary of the evidence. Int. J. Behav. Nutr. Phys. Act..

[6] Australian 24-Hour Movement Guidelines for Children and Young People (5–17 years): An Integration of Physical Activity, Sedentary Behaviour and Sleep. *Australian Government Department of Health*. https://www.health.gov.au/resources/publications/australian-24-hour-movement-guidelines-for-children-5-to-12-years-and-young-people-13-to-17-years-an-integration-of-physical-activity-sedentary-behaviour-and-sleep (2019).

[7] Tremblay MS (2016). Canadian 24-hour movement guidelines for children and youth: An integration of physical activity, sedentary behaviour, and sleep. Appl. Physiol. Nutr. Metab..

[8] Guthold, R., Stevens, G. A., Riley, L. M., & Bull, F. C. Global trends in insufficient physical activity among adolescents: A pooled analysis of 298 population-based surveys with 1.6 million participants. *Lancet Child Adolesc. Health* **4**, 23–35 (2020). 10.1016/S2352-4642(19)30323-2 PMC6919336 31761562

[9] Hanewald R (2013). Transition between primary and secondary school: Why it is important and how it can be supported. Aust. J. Teach. Educ..

[10] Morton KL (2016). School polices, programmes and facilities, and objectively measured sedentary time, LPA and MVPA: Associations in secondary school and over the transition from primary to secondary school. Int. J. Behav. Nutr. Phys. Act..

[11] Chong KH (2020). Changes in physical activity, sedentary behaviour and sleep across the transition from primary to secondary school: A systematic review. J. Sci. Med. Sport.

[12] Pearson N, Haycraft E, Johnston JP, Atkin AJ (2017). Sedentary behaviour across the primary-secondary school transition: A systematic review. Prev. Med..

[13] Kontostoli E (2021). Age-related change in sedentary behavior during childhood and adolescence: A systematic review and meta-analysis. Obes. Rev..

[14] Gaba A (2020). How do short sleepers use extra waking hours? A compositional analysis of 24-h time-use patterns among children and adolescents. Int. J. Behav. Nutr. Phys. Act..

[15] Carson V, Tremblay MS, Chaput JP, Chastin SF (2016). Associations between sleep duration, sedentary time, physical activity, and health indicators among Canadian children and youth using compositional analyses. Appl. Physiol. Nutr. Metab..

[16] Batacan RB, Duncan MJ, Dalbo VJ, Tucker PS, Fenning AS (2015). Effects of light intensity activity on CVD risk factors: A systematic review of intervention studies. Biomed Res. Int..

[17] Chastin SFM (2019). How does light-intensity physical activity associate with adult cardiometabolic health and mortality? Systematic review with meta-analysis of experimental and observational studies. Br. J. Sports Med..

[18] Farooq MA (2018). Timing of the decline in physical activity in childhood and adolescence: Gateshead Millennium Cohort Study. Br. J. Sports Med..

[19] Mitchell JA, Pate RR, Beets MW, Nader PR (2013). Time spent in sedentary behavior and changes in childhood BMI: A longitudinal study from ages 9–15 years. Int. J. Obes..

[20] Kidokoro T (2020). Physical activity and sedentary behaviour patterns among Kenyan and Japanese children: A comprehensive cross-country comparison. Int. J. Environ. Res. Public Health..

[21] Solomon-Moore E (2020). Associations of body mass index, physical activity and sedentary time with blood pressure in primary school children from south-west England: A prospective study. PLoS ONE.

[22] Judice, P. B. *et al.* Changes in physical activity and sedentary patterns on cardiometabolic outcomes in the transition to adolescence: International children's accelerometry database 2.0. *J. Pediatr.* **225**, 166–173.e1 (2020). 10.1016/j.jpeds.2020.06.018 32553870

[23] Llorente-Cantarero FJ (2020). Changes in physical activity patterns from childhood to adolescence: Genobox Longitudinal Study. Int. J. Environ. Res. Public Health..

[24] Marks J, Barnett LM, Strugnell C, Allender S (2015). Changing from primary to secondary school highlights opportunities for school environment interventions aiming to increase physical activity and reduce sedentary behaviour: A longitudinal cohort study. Int. J. Behav. Nutr. Phys. Act..

[25] Elmesmari R, Martin A, Reilly JJ, Paton JY (2018). Comparison of accelerometer measured levels of physical activity and sedentary time between obese and non-obese children and adolescents: A systematic review. BMC Pediatr..

[26] Psaltopoulou T (2019). Prevention and treatment of childhood and adolescent obesity: A systematic review of meta-analyses. World J. Pediatr..

[27] Barbosa Filho, V. C. *et al*. Promoting physical activity for children and adolescents in low- and middle-income countries: An umbrella systematic review: A review on promoting physical activity in LMIC. *Prev. Med.* **88**, 115–126 (2016). 10.1016/j.ypmed.2016.03.025 27068650

[28] Kaushal N, Rhodes RE (2014). The home physical environment and its relationship with physical activity and sedentary behavior: A systematic review. Prev. Med..

[29] Jaeschke L (2017). Socio-cultural determinants of physical activity across the life course: A 'Determinants of Diet and Physical Activity' (DEDIPAC) umbrella systematic literature review. Int. J. Behav. Nutr. Phys. Act..

[30] Chen KY, Bassett DR (2005). The technology of accelerometry-based activity monitors: current and future. Med. Sci. Sports Exerc..

[31] Dössegger A (2014). Reactivity to accelerometer measurement of children and adolescents. Med. Sci. Sports Exerc..

[32] Cain KL, Sallis JF, Conway TL, Van Dyck D, Calhoon L (2013). Using accelerometers in youth physical activity studies: A review of methods. J. Phys. Act. Health.

[33] Dobashi K (2016). Evaluation of obesity in school-age children. J. Atheroscler. Thromb..

[34] Ohkawara K (2011). Real-time estimation of daily physical activity intensity by a triaxial accelerometer and a gravity-removal classification algorithm. Br. J. Nutr..

[35] Oshima Y (2010). Classifying household and locomotive activities using a triaxial accelerometer. Gait Posture.

[36] Hikihara Y (2014). Prediction models discriminating between nonlocomotive and locomotive activities in children using a triaxial accelerometer with a gravity-removal physical activity classification algorithm. PLoS ONE.

[37] Migueles JH (2017). Accelerometer data collection and processing criteria to assess physical activity and other outcomes: A systematic review and practical considerations. Sports Med..

[38] Quené H, Bergh HV (2004). On multi-level modeling of data from repeated measures designs: A tutorial. Speech Commun..

